# Balancing the Risk of Adverse Events against the Efficacy of Immunotherapy in Advanced Thymic Epithelial Tumors

**DOI:** 10.3390/cancers15010289

**Published:** 2022-12-31

**Authors:** Birte Ohm, Wolfgang Jungraithmayr

**Affiliations:** Department of Thoracic Surgery, Medical Center–University of Freiburg, Faculty of Medicine, University of Freiburg, D-79106 Freiburg, Germany

**Keywords:** thymoma, thymic carcinoma, immunotherapy, immune checkpoint inhibition, adverse events

## Abstract

**Simple Summary:**

Treatment options for advanced thymic malignancies are limited. Immune checkpoint inhibitors are effective against advanced thymic epithelial tumors, but the treatment is associated with an elevated risk for adverse events. In this article, we aim to highlight new insights regarding predictive markers for treatment efficacy and risk of adverse events that would allow for safe and precise clinical indications for immunotherapy in thymic malignancies.

**Abstract:**

Thymic epithelial tumors (TETs) are rare thoracic malignancies with a favorable prognosis when complete surgical resection can be achieved. Therapeutic options for advanced, irresectable, or recurrent disease are limited and currently, a therapeutic standard treatment beyond platinum-based chemotherapy is undefined. Immune checkpoint inhibitors are effective against TETs, however their use is associated with a serious risk of immune-mediated toxicity. In this article, we highlight new insights regarding markers of predictive value for both treatment efficacy and risk of adverse effects in immune checkpoint inhibitor treatment for thymic epithelial tumors.

## 1. Current Role of Immune Checkpoint Inhibition in Thymic Epithelial Tumors

Thymic epithelial tumors (TETs) are a group of rare malignancies with a yearly incidence of 1.3–3.2 per million [1,2]. They are the most frequent tumors of the prevascular mediastinal compartment [3] and are classified by their histologic origin from the cortical or medullary thymic epithelium and their lymphocytic content [4]. The subtypes differ in their biological behavior, including metastatic potential and thus their overall prognosis. According to the definition of the World Health Organization (WHO), TETs are grouped into six categories [4]: Thymomas are defined as type A, AB, B1, B2, and B3 lesions and are generally well-differentiated, seldomly show invasive growth, and are of a low malignant potential. Formerly termed type C lesions, thymic carcinomas are now defined as a separate entity of thymic epithelial tumors in the 2021 WHO classification [4]. In contrast to thymoma, thymic carcinomas often infiltrate the surrounding tissue and have a higher tendency to recur or metastasize.

In early stages of the disease and whenever imaging studies suggest complete resectability, radical surgery by thymectomy including the resection of the surrounding mediastinal fat is the treatment of choice for all thymic epithelial tumors [5]. Lymphadenectomy of the anterior mediastinal lymph nodes and the anterior cervical nodes is recommended for all thymic epithelial tumors [6,7]. For thymic carcinoma, systematic lymphadenectomy should also include the middle mediastinal and deep cervical lymph nodes [6].

The standard treatment for irresectable and advanced thymic malignancies is platinum-based chemotherapy or combined radio-chemotherapy, either in a neoadjuvant or definite treatment setting. To date, a standard regimen for progressive disease during chemotherapy remains undefined [5,8].

Several clinical trials have demonstrated that the use of immune checkpoint inhibitors in these advanced thymic malignancies can be an effective option [9,10,11,12,13]. However, an enhanced risk of immune-mediated toxicity [14] and even fatal cases of such adverse events are reported in the literature [15]. To date, the exact patient populations that are particularly prone to such adverse events remain unidentified. Thus, we aim to shed light on the most recent evidence regarding markers of predictive value for both the efficacy and safety of immunotherapy in advanced TETs.

## 2. Tumor Cell Characteristics Provide A Rationale for Immune Checkpoint Inhibitor Use in Thymic Epithelial Tumors

To escape immune surveillance, cancer cells express surface proteins that hamper T cell responses despite antigen recognition. The inhibitory pathways initiated by the surface proteins programmed death 1 ligand 1 (PD-L1) and cytotoxic T lymphocyte-associated protein 4 (CTLA-4) can be targeted therapeutically by respective immune checkpoint inhibitors. This approach revolutionized cancer therapy and has provided great clinical benefits in many solid tumor types. However, many patients fail to respond to immune checkpoint inhibition, develop resistance to these agents, or experience severe adverse events. Thus, the identification of predictive biomarkers is of great interest in order to provide a precise and safe indication for immunotherapy use.

Upon their market introduction, immunohistochemistry for PD-L1 has achieved approval as biomarkers for some indications for anti-PD-L1 or anti-PD1 therapy. To date, PD-L1 detection by immunohistochemistry is the only companion test approved by the FDA for immune checkpoint inhibition in non-small cell lung cancer (NSCLC), urothelial carcinoma, cervical cancer, and triple-negative breast cancer. Due to the mechanism underlying immune checkpoint inhibition, PD-L1 expression should be a valuable predictor of tumor response. However, this biomarker can only predict a subset of responses to immune checkpoint inhibition. Notably, PD-L1 blockade can achieve robust responses in NSCLC even for tumors with low PD-L1 expression [16]. A meta-analysis demonstrated that PD-L1 can be a biomarker of overall response rate, but does not predict overall and progression free survival in NSCLC [17].

Expression of PD-L1 in thymic epithelial tumors has been confirmed in multiple studies [18,19,20] and can be found in all subtypes of thymic malignancies. Generally, PD-L1 shows the highest expression levels in B3 thymoma and thymic carcinomas and thus correlates with the grade of tumor malignancy. Furthermore, high expression of PD-L1 correlates with a higher Masaoka-Koga tumor stage [21,22]. Chemotherapy further increases PD-L1 expression levels in thymic malignancies [23], supporting the rationale for the use of immune checkpoint inhibition as a second-line therapeutic in thymic malignancies.

CTLA-4 is preferentially expressed in advanced thymoma and can be found on both thymic epithelial cells and infiltrating lymphocytes. An overexpression of CTLA-4 in these tumors correlates with reduced survival and could thus be considered a negative prognostic factor in advanced thymoma [24].

Apart from the expression of these specific targets of immune checkpoint inhibition, the tumor mutational burden (TMB) is accepted as a major prognostic factor for treatment response. The mutational burden is defined as the number of non-synonymous single nucleotide variants in a tumor. It is hypothesized that a high mutational burden favors the generation of immunogenic neoantigens that arise from somatic mutations in the cancer genome. In malignant melanoma and NSCLC, high TMB is a known predictor for response to immune checkpoint inhibition.

In comparison to these entities, thymic malignancies are characterized by a low TMB [25]. Only few studies have further evaluated the TMB in different thymic epithelial tumors. Wang et al. demonstrated that advanced stages and an advanced pathological subtype are associated with a higher mutational burden in thymoma and that TETs with a high mutational burden show a worse long-term prognosis [26]. Next generation sequencing revealed that thymic malignancies pretreated by chemotherapy show somatic mutations in some cancer-associated genes and that these mutations are more frequent in thymic carcinomas than in thymoma [27]. An analysis of the mutational status of druggable targets such as EGFR, c-KIT, KRAS, BRAF, PDGFR, HER2, and c-MET in thymic epithelial tumors did not reveal any targetable mutations except infrequent c-KIT mutations in thymic carcinoma [28]. In the context of immunotherapy, the TMB in TETs has not yet been explored to our knowledge.

Several other patient- and tumor-dependent factors are now known to have an influence on the therapeutic efficacy of immune checkpoint inhibitor treatment. These factors have recently been reviewed by Havel et al. and further potential biomarkers are continuously explored [29]. Due to the complex interaction between tumor biology and immune effectors, it is unlikely that a single biomarker that can reliably identify all patients that would benefit from immunotherapy will ever be defined. Instead, the development of predictive models encompassing multiple variables will probably refine the use of immunotherapeutic agents in the future. Currently, the information on predictive biomarkers for immunotherapy in TETs is especially limited.

## 3. Efficacy of Anti-PD-1 and Anti-PD-L1-Antibody Therapy in Advanced Thymic Malignancies

Due to the high levels of PD-L1 expression, a high efficacy of Anti-PD-1 or Anti-PD-L1 therapy should be expected in thymic malignancies. The use of Anti-PD-1-antibody in advanced thymic carcinoma was first described in 2016 [30]. To date, the efficacy of these agents has been evaluated in multiple prospective clinical trials [9,10,11,12,13].

In the largest one of these trials, Giaccone et al. evaluated the treatment with the Anti-PD-1-Antibody Pembrolizumab in 40 patients with advanced thymic carcinoma after progression despite chemotherapeutic treatment [10]. The overall response rate was 22.5% and one patient achieved complete remission. Eight patients achieved partial remission while stable disease was seen in 21 patients [10].

Similar response rates were reported by a Korean study enrolling 33 patients with progressive thymic malignancies despite chemotherapy [9]. While the majority of patients suffered from thymic carcinoma, seven patients suffering from thymoma were also enrolled. Out of these, four patients showed type B2 thymoma, two patients presented with type B3 thymoma, and one patient showed B2/3 thymoma. The study reported an overall response rate of 28.5% for advanced thymoma. Here, two patients reached partial remission while stable disease was reported for five patients. In the patients suffering from thymic carcinoma, an overall response rate of 19.2% could be demonstrated. A partial remission was reached in five patients while 14 patients achieved stable disease.

The use of the anti-PD-1-antibody Nivolumab in a Japanese phase II trial in patients with irresectable or recurrent thymic carcinoma did not achieve the same efficacy as reported for Pembrolizumab [12]. None of the fifteen patients enrolled in the study reached complete or partial remission. Stable disease was reported for eleven patients.

Currently, combination therapy of Nivolumab and the anti-CTLA4-antibody Ipilimumab is evaluated in advanced or recurrent B3 thymoma or thymic carcinoma in the NIVOTHYM trial [13]. In the trial cohort receiving Nivolumab monotherapy, an overall response rate of 12% was reported [13].

A phase I trial evaluated the use of the anti-PD-L1 antibody Avelumab in seven Patients with advanced thymoma and one patient with thymic carcinoma [11]. Overall, the objective response rate was 57%. However, a confirmed response by imaging studies was only found in 29% [11]. In this trial, the best response was a partial response.

All prospective clinical trials evaluating the use of immune checkpoint inhibitors in thymic malignancies have been conducted in single-arm designs. Furthermore, a standard therapy upon disease progression during platinum-based chemotherapy remains undefined to date. A direct comparison between the above-mentioned studies is problematic due to the heterogeneity of the trial populations. However, one can conclude that immune checkpoint inhibition can achieve a reasonable control of disease when standard chemotherapeutic regimens have failed. Table 1 provides a summary of the efficacy endpoints reached in clinical trials employing immune checkpoint inhibition in advanced thymic malignancies.

## 4. Risk of Immune-Mediated Adverse Events in Immunotherapy for TETs

As immune checkpoint inhibition results in an overall stimulation of the T cell immune response, its use is associated with immune-related adverse events (irAEs) due to the activation of T cell responses targeting self-antigens. While the rate of severe irAEs is reported below 10% in most solid tumors [31], the treatment of thymic malignancies is associated with an enhanced risk of severe irAEs [10]. These irAEs encompass myasthenia gravis, myocarditis, hepatitis, and myositis and usually respond well to corticosteroid treatment [9,10]. Importantly, the rate of irAEs differs between thymic carcinoma and thymoma.

In thymic carcinoma, approximately 15% of the patients treated with Pembrolizumab experience severe irAEs [9,10]. Treatment with Nivolumab similarly resulted in 13% of patients developing severe irAEs leading to hospitalization in the PRIMER study [12].

Even higher rates of irAEs are reported for immunotherapy in thymoma. Cho et al. reported that five out of seven patients (71.4%) treated with pembrolizumab experienced irAEs requiring high-dose corticosteroid treatment [9]. Avelumab treatment also caused irAEs in five out of seven patients (71.4%) [11]. Some of these patients even experienced a simultaneous co-occurrence of several different irAEs [9,11]. Table 2 summarizes the incidence of severe AEs in the available clinical trials employing immune checkpoint inhibition in thymic epithelial tumors.

Based on these observations, one may conclude that thymic malignancies pose a significant risk for the development of unspecific T cell responses upon immunotherapy. This is especially relevant in thymoma. However, the mechanistic base of these observations remains unclear.

In an effort to define predictive biomarkers for irAEs, Rajan et al. reported that patients with thymoma who developed irAEs had a higher T cell receptor diversity, lower B cell counts, and lower levels of regulatory T cells in their peripheral blood before treatment initiation [11]. Furthermore, B cell lymphopenia and pre-existing anti-acetylcholine receptor autoantibodies were reported as risk factors for myositis during avelumab treatment for thymoma [32]. Notably, PD-L1 expression did not correlate with risk of irAEs in either thymic carcinoma or thymoma [9,10].

## 5. Implications of the Tumor Immune Microenvironment of Thymic Epithelial Tumors for Efficacy and Safety in Immunotherapy

Immune checkpoint inhibition enhances the immune response mounted towards tumor cells and takes advantage of the immune cell infiltrate at the tumor site. Given the difference between squamous cell thymic carcinoma and thymoma regarding irAE risk, it seems likely that the composition of this immune microenvironment is mechanistically critical for both, the overall response to as well as the risk for adverse events in immune checkpoint inhibition. In the following section, we aim to discuss the relevance of different immune effectors of the tumor immune microenvironment and their occurrence in thymic malignancies.

T cells are the primary effector cells in the anti-tumor response upon immune checkpoint inhibition, as these therapeutics were designed to invigorate their responsiveness. Not surprisingly, several studies demonstrated a more favorable response to PD-1/PD-L1 blockade in tumors showing a higher infiltration of CD8+ cytotoxic T cells [33]. A higher density of such cytotoxic CD8+ T cells in the tumor core and the invasive margins was shown to correlate with an increased response to PD-1/PD-L1 blockade [34].

To mount an effective anti-tumor response, cytotoxic T cells at the tumor site have to remain functional. While immune checkpoint inhibition does not increase the number of CD8+ T cells, the treatment enhances their cytolytic capacity [35] and an increased expression of cytotoxicity-related genes can be observed upon both PD-1 blockade and anti-CTLA4-Antibody treatment [36,37].

While CD8+ T cells are primarily known for their direct cytotoxic effect on tumor cells, CD4+ T cells mostly aid in anti-tumor immunity in an indirect manner. In a murine model of sarcoma, it was demonstrated that the recognition of tumor cell peptides presented on MHC II by CD4+ T helper cells was crucial for the generation of a functional CD8+ T cell response in immune checkpoint inhibition [38]. However, there is also evidence of intratumoral cytotoxic CD4+ T cells equipped with granzyme and perforin, whose activity can be enhanced by immunotherapy [39,40].

Not all tumor-infiltrating T cells promote an anti-tumor response. Regulatory T cell subsets (Tregs) characterized by the expression of CD4 and FoxP3 generally dampen the anti-tumor immune response. Importantly, this T cell subset also expresses CTLA-4 constitutively and thus is targetable by CTLA-4 blockade in cancer therapy [41]. The presence of Tregs has been associated with response to CTLA-4 inhibition and treatment efficacy was demonstrated to depend on Treg depletion [42]. However, the use of immune checkpoint inhibitor treatment does not always reduce the effects of regulatory T cells [43]. Indeed, PD-1 blockade was shown to increase Treg infiltrates and can thus promote tumor hyperprogression [44].

As the thymus aids in T cell differentiation as a primary lymphoid organ, it is not surprising that T cells of different maturation states make up the most abundant cell population of the tumor immune microenvironment in TETs.

In type A/B/B1 and B2 thymomas, immature T cells expressing both CD4 and CD8 are the most abundant type of cells [45,46]. Based on their immunophenotype, these cells closely resemble normal thymocytes. Thus, the microenvironment in these lower-grade tumors mimics the functionally normal thymus. In contrast, type B3 thymomas and thymic carcinomas are characterized by an immune cell infiltrate of terminally differentiated T cells expressing either CD4 or CD8 [45,46], which may be more prone to mount an effective antitumoral response. Immature T cells in type A/B/B1 and B2 thymomas carry the potential to mature and thus generate auto-reactive T cell responses [47] and are therefore associated with a higher rate of paraneoplastic syndromes. In addition, thymoma shows a reduced expression of Foxp3 compared to normal or hyperplastic thymic tissue [48] and this lack of Tregs may contribute to autoimmune responses.

Considering the T cell infiltrate usually found in thymoma, B3 thymomas and thymic carcinoma should be the two entities that provide the most beneficial risk-benefit-assessment for an immunotherapeutic approach. The high prevalence of mature T cells in these tumor types provides cellular targets for a successful enhancement of the antitumoral immune response. The increased risk for irAEs in thymoma of type A/B/B1 and B2 could be explained by their immune microenvironment including a plethora of immature T cells that may evade physiological selection mechanisms.

B lymphocytes can be found in micronodular thymic neoplasms with lymphoid hyperplasia [49]. They can be both present in thymoma as well as thymic carcinoma. Typically, B cells form aggregates reminiscent to germinal centers. Oftentimes, B cells are found in the thymic tissue of patients suffering from autoimmune disease such as Myasthenia gravis [50].

The precise role of B cells in immunotherapy still remains elusive. It seems likely that the intratumoral presence of memory-like B cells and plasmablasts may qualify as a predictor of response [51]. B cells are also critical for the generation of tertiary lymphoid organs (TLOs). These lymphocyte aggregates resemble germinal centers and are believed to promote anti-tumoral immune responses. Indeed, their presence has been associated with a higher response rate to immune checkpoint inhibition [52].

In thymic epithelial tumors, B cellular infiltrates are closely associated with the occurrence of paraneoplastic autoimmune responses. Therefore, the enhanced presence of intratumoral B cells should urge caution regarding the use of immunotherapy in these tumors. In general, pre-existing autoimmune conditions are associated with flares and an increased rate of irAEs during immune checkpoint inhibitor therapy [53]. The most frequent autoimmune condition in thymic epithelial tumors is myasthenia gravis. As this condition can also be induced by immune checkpoint inhibition [54], the use of these agents in thymic malignancies accompanied by myasthenic symptoms should possibly be avoided. Unfortunately, those thymomas preferentially targetable by immune checkpoint inhibition due to a high PD-L1 expression and high counts of CD8+ lymphocytic infiltrates are often associated with myasthenia gravis [21]. Mechanistically, the intratumoral presence of autoreactive T cells can enhance PD-L1 expression by their release of interferon-gamma (IFN-γ) [55]. In both clinical trials evaluating immune checkpoint inhibition in thymoma, active autoimmune disease was listed as an exclusion criterion [9,11].

## 6. Conclusions

**Figure 1:**
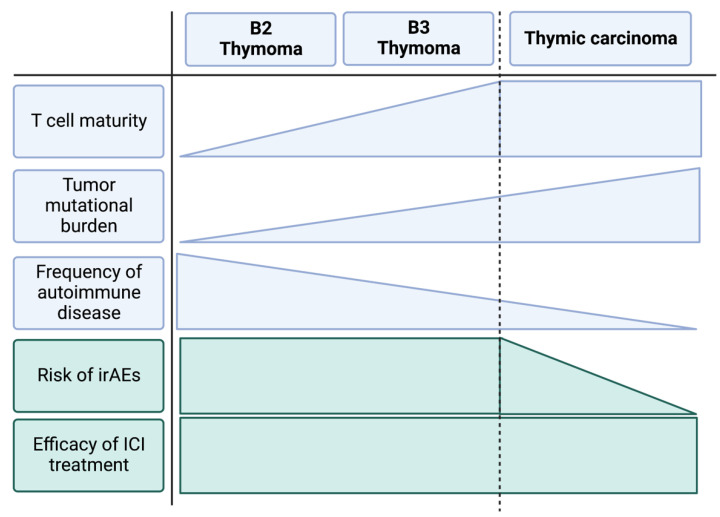
Schematic depiction of tumor characteristics of the most frequent types of advanced thymic epithelial tumors and their relation to irAE risk and efficacy of immune checkpoint inhibitor treatment. We suggest that the higher rate of irAEs in thymoma is related to the intratumoral presence of immature T cells and their association with autoimmune reactions. Thymic carcinoma presents with mature T cell infiltrates and is not associated with autoimmunity. Clinical trials demonstrate a lower risk for irAEs during immune checkpoint inhibition for thymic carcinoma compared to advanced thymoma while reporting a similar efficacy.

Systemic treatment options for advanced thymic epithelial tumors beyond platinum-based chemotherapy are limited. As TETs show a consistently increased expression of immune checkpoints in advanced stages, immune checkpoint inhibition potentially provides a reasonable control of the disease and can achieve a clinical response in approximately 20% of all cases. However, the treatment is associated with high rates of irAEs—especially in patients suffering from thymoma. In order to provide a safe clinical use of immune checkpoint inhibitors in thymic epithelial tumors, predictors of both response and adverse events need to be identified. Apart from tumor cell characteristics, such as the expression of the targetable immune checkpoints or mutational burden, an in-depth analysis of the tumor immune microenvironment may provide clues to the clinical response of the individual patient. From what is known about the general histomorphologic composition of thymic epithelial cells, one may speculate that thymic carcinoma and advanced type B3 thymoma should be the entities of choice regarding the use of immune checkpoint inhibition in thymic epithelial tumors. These tumors show a comparatively high malignant and invasive potential and are characterized by an infiltration of mature T lymphocytes. On the other hand, other subtypes of thymoma commonly show infiltrates of immature T cells that may provide the base for autoimmune phenomena and therefore irAEs during immune checkpoint inhibitor treatment. Figure 1 highlights these risk factors regarding the unique tumor immunity of thymoma and thymic carcinoma. Their exploration in further clinical trials may provide the key to optimal decision-making in immunotherapy for advanced thymic malignancies.

## Figures and Tables

**Table 1:** Clinical trials evaluating the efficacy of immune checkpoint inhibition in thymic epithelial tumors. ORR = overall response rate, DCR = disease control rate, mPFS = median progression free survival, mOS = median overall survival, nr = not reported, - = endpoint not reached.

Reference	Therapeutic Agent	Tumor Entity	Number of Patients	DCR (%)	ORR (%)	mPFS (Months)	mOS (Months)
Giaccone et al. 2018	Pembrolizumab	Thymic carcinoma	40	75	22.5	4.2	24.9
Cho et al. 2018	Pembrolizumab	Thymic carcinoma	26	73	19.2	6.1	14.5
		Thymoma	7	100	28.6	6.1	-
Katsuya et al. 2019	Nivolumab	Thymic carcinoma	15	73.3	0	3.8	14.1
Rajan et al. 2019	Avelumab	Thymic carcinoma	1	100	0	nr	nr
		Thymoma	7	85.7	29	nr	nr

**Table 2:** Rates of Grade 3–4 adverse events (AEs) (according to the Common Terminology Criteria for Adverse Events) in clinical trials evaluating immune checkpoint inhibition in thymic epithelial tumors. Studies including patients with Thymoma report higher rates of severe AEs in these patients.

Reference	Therapeutic Agent	Tumor Entity	Number of Patients	AEs Grade 3–4 (% of Patients)
Giaccone et al. 2018	Pembrolizumab	Thymic carcinoma	40	15
Cho et al. 2018	Pembrolizumab	Thymic carcinoma	26	15.4
		Thymoma	7	71.4
Katsuya et al. 2019	Nivolumab	Thymic carcinoma	15	20
Rajan et al. 2019	Avelumab	Thymic carcinoma	1	-
		Thymoma	7	71.4
